# Case Report: A Challenging Localization of a Pulmonary Ectopic ACTH-Secreting Tumor in a Patient With Severe Cushing’s Syndrome

**DOI:** 10.3389/fendo.2021.687539

**Published:** 2021-07-09

**Authors:** Andreea Liliana Serban, Lorenzo Rosso, Paolo Mendogni, Arianna Cremaschi, Rita Indirli, Beatrice Mantovani, Mariagrazia Rumi, Massimo Castellani, Arturo Chiti, Giorgio Alberto Croci, Giovanna Mantovani, Mario Nosotti, Emanuele Ferrante, Maura Arosio

**Affiliations:** ^1^ Endocrinology Unit, Fondazione IRCCS Ca’ Granda Ospedale Maggiore Policlinico di Milano, Milan, Italy; ^2^ Thoracic Surgery and Lung Transplant Unit, Fondazione IRCCS Ca’ Granda Ospedale Maggiore Policlinico, Milan, Italy; ^3^ Department of Pathophysiology and Transplantation, University of Milan, Milan, Italy; ^4^ Department of Clinical Sciences and Community Health, University of Milan, Milan, Italy; ^5^ Hepatology Unit, Ospedale San Giuseppe Multimedica Milan, Milan, Italy; ^6^ Department of Nuclear Medicine, Fondazione IRCCS Ca’ Granda Ospedale Maggiore Policlinico di Milano, Milan, Italy; ^7^ Department of Biomedical Sciences, Humanitas University, Milan, Italy; ^8^ Department of Biomedical Sciences, IRCCS Humanitas Research Hospital, Milan, Italy; ^9^ Division of Pathology, Foundation IRCCS Ca’ Granda Ospedale Maggiore Policlinico, Milan, Italy

**Keywords:** Cushing’s syndrome, ectopic ACTH syndrome, pulmonary carcinoid, rectal carcinoma, hypercortisolism

## Abstract

**Background:**

Ectopic adrenocorticotropic syndrome (EAS) is a rare cause of endogenous ACTH-dependent Cushing’s syndrome, usually associated with severe hypercortisolism as well as comorbidities. Tumor detection is still a challenge and often requires several imaging procedures. In this report, we describe a case of an ectopic ACTH secretion with a misleading localization of the responsible tumor due to a concomitant rectal carcinoma.

**Case presentation:**

A 49-year-old man was referred to our Endocrinology Unit due to suspicion of Cushing’s syndrome. His medical history included metastatic rectal adenocarcinoma, diagnosed 5 years ago and treated with adjuvant chemotherapy, radiotherapy and surgical resection. During follow-up, a thoracic computed tomography scan revealed two pulmonary nodules located in the superior and middle lobes of the right lung with a diameter of 5 and 10 mm, respectively. However, these nodules remained radiologically stable thereafter and were not considered relevant. All biochemical tests were suggestive of EAS (basal ACTH levels: 88.2 ng/L, nv 0–46; basal cortisol levels: 44.2 µg/dl, nv 4.8–19.5; negative response to CRH test and high dose dexamethasone suppression test) and radiological localization of the ectopic ACTH-secreting tumor was scheduled. The CT scan revealed a dimensional increase of the right superior lung nodule (from 5 to 12 mm). [^68^Ga]-DOTA-TOC PET/CT scan was negative, while [^18^F]-FDG-PET/CT showed a tracer accumulation in the superior nodule. After a multidisciplinary consultation, the patient underwent thoracic surgery that started with two atypical wedge resections of nodules. Frozen section analyses showed a neuroendocrine tumor on the right middle lobe nodule and a metastatic colorectal adenocarcinoma on the superior lesion. Then, a right superior nodulectomy and a right middle lobectomy with mediastinal lymphadenectomy were performed. The final histopathological examination confirmed a typical carcinoid tumor, strongly positive for ACTH. A post-surgical follow-up showed a persistent remission of Cushing’s syndrome.

**Conclusions:**

The present report describes a case of severe hypercortisolism due to EAS not detected by functional imaging methods, in which the localization of ACTH ectopic origin was puzzled by a concomitant metastatic rectal carcinoma. The multidisciplinary approach was crucial for the management of this rare disease.

## Introduction

Cushing’s syndrome (CS) is caused by a chronic exposure to supraphysiological levels of glucocorticoids leading to several comorbidities and high mortality if not adequately treated (1). An ectopic adrenocorticotropic (ACTH) syndrome (EAS) is an infrequent form of endogenous ACTH-dependent CS (2), usually associated with intense hypercortisolism (3) as well as severe comorbidities such as hypokalemia, diabetes mellitus, infections and vertebral fractures (4–7). Since the recommended first line treatment of EAS is the surgical removal of the ectopic ACTH-secreting tumor (EAT) (8), its prompt localization is crucial. However, the first imaging study succeeds in identifying the EAT in only 50–60% of cases, while the ectopic origin of ACTH may remain occult for several years in up to one-fifth of patients (9). A failure in the localization of the ACTH ectopic source requires additional imaging procedures, second-line therapies (ranging from pharmacological treatment to bilateral adrenalectomy) and a close follow up.

Here we describe the case of a severe CS due to ectopic ACTH secretion with a misleading localization of the responsible tumor due to a concomitant rectal carcinoma.

## Case Description

A 49-year-old Caucasian man was referred to our Endocrinology Unit in June 2020 from the Hepatology Unit with suspicion of CS. The patient reported a recent occurrence of severe backache and progressive muscular weakness, leading to gait impairment and the need to use crutches. His past medical history was relevant for a left knee chondroblastoma, diagnosed and surgically treated at 14 years of age, and a rectal adenocarcinoma with liver metastasis, diagnosed in 2015 and treated with adjuvant chemotherapy, radiotherapy and surgical resection. Thereafter the patient underwent a regular oncologic follow-up and in June 2017, a thoracic computed tomography (CT) scan revealed two pulmonary nodules located in the superior and middle lobes of the right lung with a diameter of 5 and 10 mm, respectively. However, since these two nodules remained stable during radiological follow-up, including CT scans in 2018 and 2019, and did not show fluorodeoxyglucose ([^18^F]-FDG) uptake at positron emission tomography (PET) performed in September 2017, they were not considered relevant.

The patient also had arterial hypertension treated with monotherapy, diagnosed at the age of 34, diabetes mellitus diagnosed 3 months earlier, was in diet therapy and had a hepatitis C virus infection found in 2016 and had it subsequently eradicated through an antiviral treatment with Ombitasvir/Dasabuvir plus weight-based Ribavirin.

Physical examination showed the patient being overweight (BMI: 26.6 kg/m^2^) with central adiposity, a moon face and a buffalo hump. The patient also presented proximal miopathy and scattered bruises, while no striae rubrae was present. The blood pressure was at 170/120 mmHg and the fasting capillary glycemia was at 310 mg/dl. The other biochemical features are summarized in **Table 1**.

**Table 1:** Biochemical features at presentation and laboratory diagnostic work-up.

	Patient’s value	Reference interval
Hemoglobin (g/dl)	15.5	13.5–17.5
Leucocytes (10^9^/L)	10.05	4.8–10.8
Neutrophyles (10^9^/L)	8.07	1.5–6.5
PCR (mg/dl)	5.4	<0.5
APTT (ratio)	0.8	0.86–1.2
Glycemia (mg/dl)	291	70–110
HbA1c (mmol/mol)	71	20–42
Na^+^ (mEq/L)	141	135–145
K^+^ (mEq/L)	2.5	3.3–5.1
ALT (U/L)	269	9–59
AST (U/L)	74	10–35
GGT (U/L)	883	8–61
TGL (mg/dl)	584	<150
Total cholesterol (mg/dl)	196	<190
HDL cholesterol (mg/dl)	26	
Basal cortisol (8.00 AM, µg/dl)	44.2	4.8–19.5
Basal ACTH (8.00 AM, ng/L)	88.2	0–46
Cortisol (µg/dl) after 1 mg dexamethasone suppression test	44.8	<1.8
Urinary free cortisol (µg/dl)	770	<60
LH (mIU/L)	<0.3	1.7–8.6
Testosterone (µg/L)	0.59	2.8–8.4
TSH (mIU/L)	0.47	0.28–4.3
FT4 (ng/L)	6.4	8–17
PRL (µg/L)	27.8	1.7–16
IGF1 (µg/L)	121	50–200
**Biochemical tests for diagnosis of EAS**		
CRH stimulation test: morning basal ACTH: 72 ng/ml → ACTH peak: 80.4 (+11%) morning basal cortisol: 39 mcg/dl → cortisol peak: 42.9 mcg/dl (+10%)		
High dose Dexamethasone Suppression Test cortisol: 37.6 mcg/dl (−3.5% *vs* basal)		

**Figure 2:**
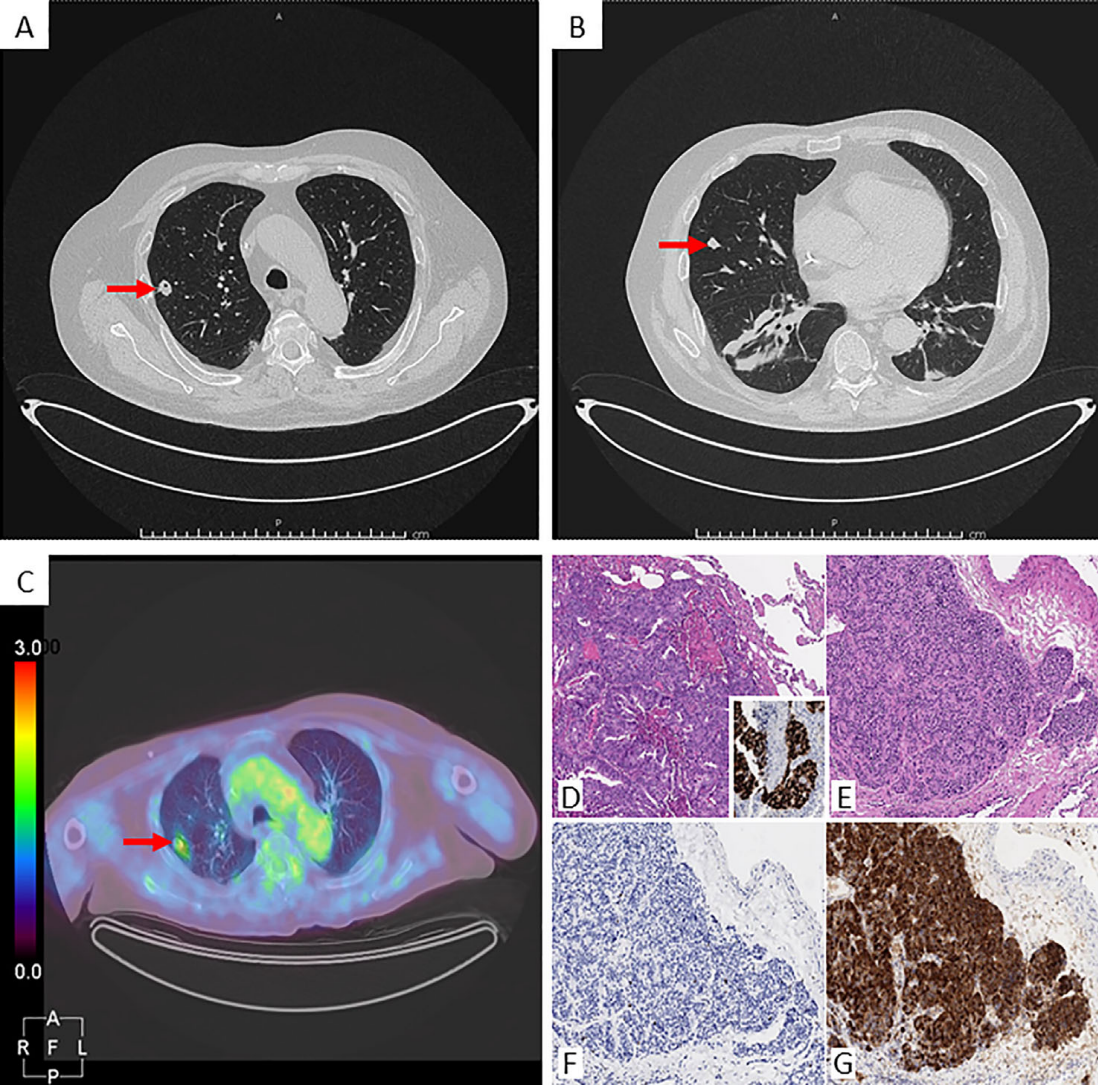
Radiological images and histological panel of the lung nodules. Chest CT scan: **(A)** nodule in the right superior lobe (arrow), axial projection; **(B)** nodule in the right middle lobe (arrow), axial projection. [^18^F]-FDG-PET/CT: **(C)** uptake of the right superior nodule (arrow), axial projection. Histological panel: the nodule from the superior right lobe (**D** Hematoxylin-Eosin, 100x) consists of a metastatic carcinoma, with its colo-rectal primitivity confirmed upon CDX2 positivity at immunohistochemistry (inset). Detail from the middle lobe nodule lobe (**E** Hematoxylin-Eosin, 100x) depicts an epithelial neoplasm growing in an organoid fashion, composed of bland-looking cells with low mitotic rate and proliferative index (**F** Ki67/Mib1, 100x), estimated within a 1-2% range, and featuring intense positivity for ACTH at immunohistochemistry (**G** 100x).

The diagnostic work-up included four major steps: 1) Biochemical confirmation of CS; 2) Classification as ACTH-dependent CS; 3) Differential diagnosis between Cushing’s Disease (CD) and EAS; and 4) Localization of ACTH-secreting tumor and evaluation of its extension. The biochemical diagnosis was rapidly posed as all the tests were suggestive of EAS (**Table 1**) (10, 11). The pituitary magnetic resonance imaging (MRI) revealed a small pituitary adenoma of 1.9 mm, which was interpreted as irrelevant in the final diagnosis. Bilateral inferior petrosal sinus sampling was not performed, as the clinical and the biochemical findings were considered sufficient to support the diagnosis of EAS. A radiological localization of the ectopic ACTH-secreting tumor was scheduled. The CT scan revealed a significant increase of the lung nodule located in the right superior lobe (from 5 to 12 mm), while the nodule in the right middle lobe was unchanged (**Figure 2**). However, a [^68^Ga]-DOTA-TOC PET/CT scan did not show any pathologic tracer accumulation. On a [^18^F]-FDG-PET/CT (capillary glycemia <150 mg/dl before examination), the right superior nodule took-up the radiopharmaceutical with a maximum standardized uptake value (SUV) of 2.4, while the other pulmonary nodule did not accumulate [^18^F]-FDG (**Figure 2**). It must be underlined that the smaller nodule could have been close to the spatial resolution limit of the PET scanner.

The assessment of the CS-associated comorbidities revealed multiple vertebral and costal fractures, the former accounting for the patient’s back pain; rapidly worsening glucose control, which required basal-bolus insulin therapy; severe hypokalemia and refractory hypertension which needed a progressive increase of canrenone of up to 300 mg/day in addition to three other antihypertensive drugs (**Table 2**).

**Table 2:** Associated comorbidities: treatment and progression.

Comorbidities	Medical treatment		3 months after surgical cure
Hypertension	Perindopril, Amlodipin, Bisoprolol	+ Metyrapone 1,000 mg/day	Improved
Hypokalemia	Canrenone, Potassium Chloride		Remitted
Diabetes mellitus	Basal-bolus Insulin		Remitted
Dyslipidemia	Diet		Normalized
NASH	Diet		Improved
Osteoporosis with vertebral and costal fractures	Zoledronate, Calcium Carbonate, Cholecalciferol, Orthopaedic brace		–
Mixed anxiety-depressive disorder	Escitalopram Bromazepam		Improved
Mandibular abscess	Amoxicillin + Clavulanic Acid, Dental Treatment		Remitted
Increased thromboembolic risk*	Enoxaparin		APTT normalised
Central hypogonadism	Not treated		Remitted
Central hypothyroidism	Not treated		Remitted

*Reduced APTT, reduced mobility, infection, very high UFC levels.

**Figure 1:**
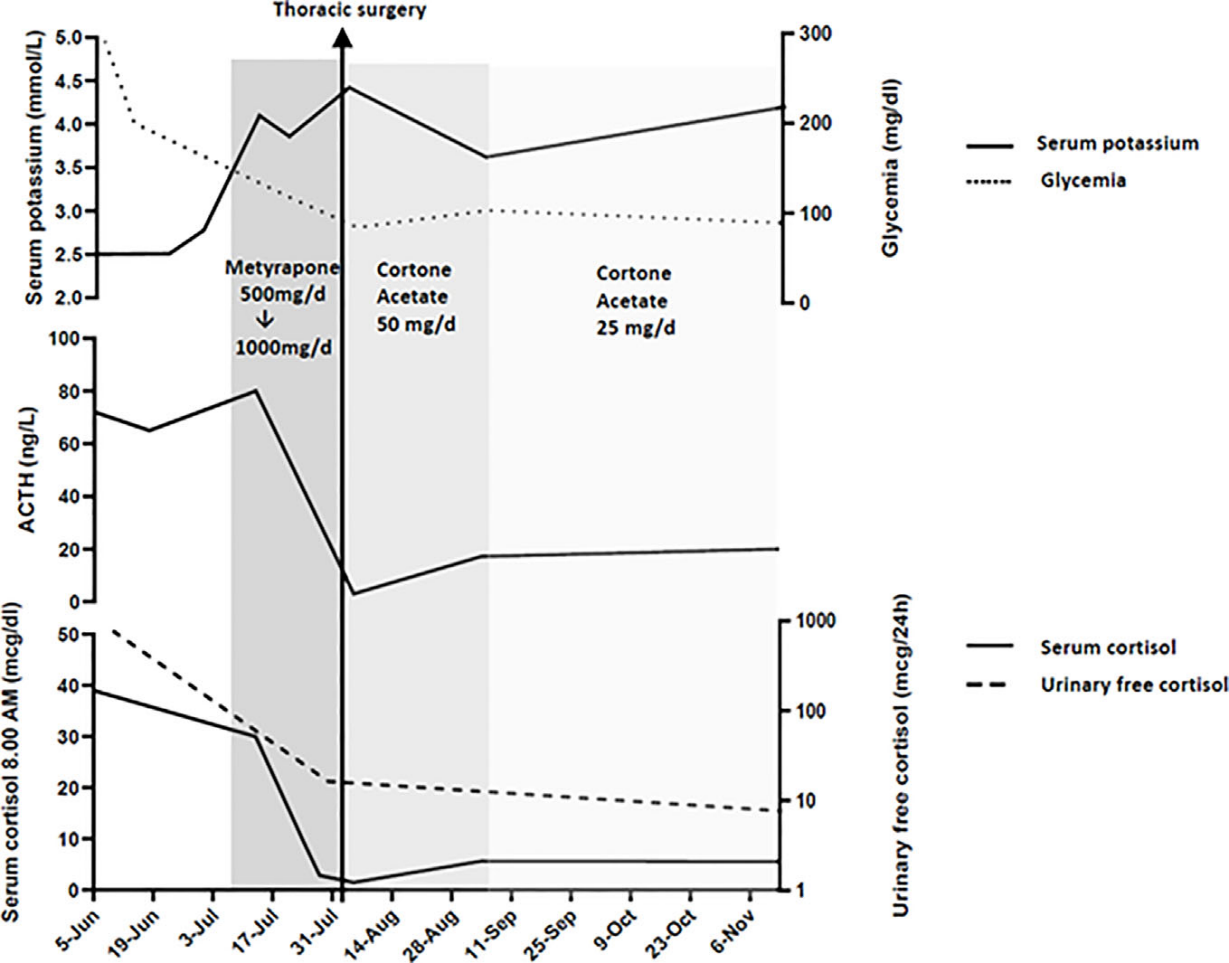
Serum cortisol, ACTH, glycemia, potassium and urinary free cortisol levels at presentation, during medical therapy and after thoracic surgery.

Immediately after the laboratory and radiological assessment, medical therapy with metyrapone, a steroidogenesis enzyme inhibitor, was started. Due to severe and refractory hypokalemia and hypertension, the initial dose was at 500 mg/day, which quickly increased to 1,000 mg/day. This dose allowed a rapid and important reduction of basal cortisol levels and a normalization of free urinary cortisol (**Figure 1**).

In the meantime, the case was discussed in a multidisciplinary team that included an endocrinologist, a thoracic surgeon, an anaesthesiologist, a neurosurgeon, and a radiologist. After that, it was decided to refer the patient to a thoracic surgery with the aim to analyze the pulmonary nodules through frozen section and then proceed with thoracic surgery, according to the results. The patient was informed by the multidisciplinary team on the procedure and its associated risks, including the possible need for a chronic oxygen therapy because of his low pulmonary function. The patient accepted the surgery and pulmonary rehabilitation, in addition to continuous-positive airway pressure (CPAP) therapy, which was immediately started. One week later, the patient underwent a minimally invasive thoracic surgery. Surgical procedure started with two atypical wedge resections of nodules. Frozen section analyses resulted positive for neuroendocrine tumor on right middle lobe nodule and for metastatic colorectal adenocarcinoma on the superior lesion. For oncological reasons a right middle lobectomy and a mediastinal lymphadenectomy were performed. The postoperative course was uneventful and the patient was discharged on post-operative day 4 in good clinical condition. The final histopathological examination confirmed the frozen section procedure results: a colorectal carcinoma metastasis in the right superior pulmonary nodule and a typical carcinoid tumor (according to the WHO 2015 classification), strongly positive for ACTH immunostaining, in the middle lobe (**Figure 2**). After surgery, low levels of morning plasmatic ACTH and cortisol (ACTH <5 pg/ml, cortisol 1.52 µg/dl) confirmed EAS remission. In the following days, the general clinical status of the patient significantly improved, thus allowing the withdrawal of insulin therapy, canrenone, potassium chloride and the reduction of anti-hypertensive drugs dosage (**Table 2**). Three months after surgery, a biochemical evaluation showed a persistent remission of the disease and the patient was able to walk without crutches.

## Patient Perspective

“I remember my first endocrinological visit at Fondazione Ca’ Granda, Ospedale Maggiore Policlinico, I was feeling tired, very anxious, also depressed, with mood swings and I was having difficulties to concentrate. My back was aching very hard and I was able to walk only a few steps with two crutches and my face was swollen and reddish. My symptoms began three four months before and worsened quickly. The weeks after the surgical procedure I was feeling very tired, my appetite lacked, but my mind was clear and I was feeling more optimist. After three months my back still hurts, but less than before and I can easily walk with one crutch, so now I’m autonomous. My appetite is normal, I feel energetic, my mind is clear and I have begun to do small jobs. Overall, I can say that my quality of life significantly improved after the surgical intervention.”

## Discussion

The present case report describes an unusual association of a lung carcinoid tumor responsible for a severe EAS with a metastatic rectal cancer that puzzled the localization of ectopic ACTH source. Although the state of intense hypercortisolism is not precisely defined in the literature, it was seen that the CS complications are more frequent and rapidly evolving when UFC is increased five times above the upper limit of normal range (ULN) (12). In these cases, many patients need urgent control of hypercortisolism. Our patient had UFC levels approximately 11 times above ULN and presented rapid occurrence and worsening of several CS comorbidities already described in the literature (1).

As more than a half of ectopic ACTH-secreting tumors are located in the chest (45% pulmonary NET, 6.5% thymic NET) (9, 13) and taking into account patient’s medical history, imaging investigation started with a thorax CT scan that revealed a significant increase of the right superior lung nodule in the last year. Overall, the CT scan has a sensitivity of 82% (77–85%) when used to detect different types of NET (14). In the case of EAS, a systematic review conducted by Isidori and colleagues showed that only in a half of cases cross-sectional imaging was positive at presentation, while in the rest of cases the tumor was detected during follow-up, also through functional imaging (29% cases), or it was never found (18%) (9). In our particular case, considering also the previous diagnosis of rectal carcinoma, the detection of two distinct nodules at the thorax CT made the origin of ectopic ACTH secretion unclear. Therefore, the patient underwent a [^68^Ga]-DOTA-TOC PET/CT scan, that resulted negative, and a [^18^F]-FDG-PET/CT scan that showed a radiopharmaceutical uptake of the nodule located in the superior lobe of right lung. The second nodule was not reported as [^18^F]-FDG avid, although the small size lowered the sensitivity of the examination. The performance of [^68^Ga]-DOTA-TOC PET/CT and [^18^F]-FDG-PET/CT for the detection of EAT is still debated. [^68^Ga]-DOTA-TOC PET/CT is usually preferred in occult tumors, that are often non-metastatic and well-differentiated NETs. In fact, the slow growth rate of these tumors can determine a negative result to [^18^F]-FDG-PET/CT (15), which is more useful to characterize the behavior of tumor detected at CT, since a positive result is more frequently associated with atypical or aggressive NETs (16).

[^68^Ga]-DOTATOC PET/CT provides a high sensitivity (88–93%) and specificity (88–95%) for the diagnosis of carcinoid tumor (14), although a recent systematic review reported a significantly lower overall sensitivity of 76.1% for EAS (17). Furthermore, its detection rate may be lower in high grade NET. A study conducted by Binderup and colleagues showed that in neuroendocrine tumors with proliferation index >15%, somatostatin receptor scintigraphy reached a sensitivity of only 69%, much lower than 92% of [^18^F]-FDG-PET/CT (18). It must be underlined that somatostatin receptor scintigraphy has a lower sensitivity when compared to somatostatin receptor PET and these results are underestimating the accuracy of [^68^Ga]-DOTA-TOC PET/CT (19). In a different manner, the lack of pathological uptake to [^68^Ga]-DOTA-TOC may also be related to the finding that glucocorticoids downregulate the somatostatin receptors (20, 21). Moreover, in two cases of EAS, the reduction of cortisol levels using mifepristone permitted the localization of ACTH ectopic origin with ^111^In-pentetreotide (22). Of note, at the time of imaging, the patient had extremely elevated levels of urinary free cortisol (x11 ULN, see **Table 1**).

As a whole, according to CT scan and functional imaging, we defined three possible scenarios: i. occult EAT with two lung nodules of uncertain origin (metastasis of rectal carcinoma in superior lobe)?; ii. EAT of right middle lobe with false negative [^68^Ga]-DOTA-TOC PET/CT associated with probable metastasis at superior lobe; and iii. High grade NET of right superior lobe with false negative [^68^Ga]-DOTA-TOC PET/CT associated with middle lobe indeterminate lesion.

As stated before, an indication to thoracic surgery was confirmed after a multidisciplinary discussion and motivated by the following arguments: first, the patient suffered from a severe and life-threatening form of CS; second, metyrapone therapy is able to inhibit adrenal steroidogenesis but it does not target the primary cause of cortisol excess and may have various side effects such as hypertension, hypokalaemia, and adrenal insufficiency (23); and third, the biopsy of two pulmonary nodules was already indicated in the context of previous diagnosis of rectal carcinoma.

The frozen section examination revealed an EAT in the middle lobe of the right lung and a rectal carcinoma metastasis in the right superior lobe. An atypical resection of the right superior pulmonary lobe for the rectal metastasis and a middle lobectomy lymphadenectomy for the ACTH positive carcinoid tumor were performed accordingly. Then, in the present case the pulmonary carcinoid tumor was not detected in either functional imaging methods.

In conclusion, the present report describes a case of severe hypercortisolism due to EAS in which the localization of ACTH ectopic origin was puzzled by a concomitant metastatic rectal carcinoma. The biochemical diagnosis of EAS was instrumental in the reassessment of apparently benign lung nodules and the past history of metastatic rectal cancer imposed a rapid surgical exploration of both lesions. A multidisciplinary approach as well as a clear and close communication with the patient played a crucial role in the management of this case.

## Data Availability Statement

The raw data supporting the conclusions of this article will be made available by the authors, without undue reservation.

## Ethics Statement

Written informed consent was obtained from the individual(s) for the publication of any potentially identifiable images or data included in this article.

## Author Contributions

AS, GM, EF, MA, MR, MC, and ACh: diagnostic approach of the case and writing the paper. AS and GC: editing images. ACr, RI, and BM: sample collection and preparation and patient follow-up. LR, PM, and MN: surgical treatment of the patient and writing the paper. GC: histopathological examination. All authors contributed to the article and approved the submitted version.

## Funding

This work was supported by Ricerca Corrente Funds from the Italian Ministry of Health.

## Conflict of Interest

The authors declare that the research was conducted in the absence of any commercial or financial relationships that could be construed as a potential conflict of interest.
